# Using vignettes to understand variations in patient screening and recruitment

**DOI:** 10.1186/1745-6215-16-S2-O22

**Published:** 2015-11-16

**Authors:** Elaine McColl, Paul Hilton, Cath Brennand

**Affiliations:** 1 Newcastle University, Newcastle upon Tyne, UK

## 

The INVESTIGATE-I study was designed to inform a future definitive randomised trial of invasive urodynamic testing compared to basic clinical assessment with non-invasive tests, prior to surgical treatment in women with stress urinary incontinence or stress predominant mixed urinary incontinence. In a pilot RCT, women from seven participating sites were screened, consented and randomised. Overall, 771 patients were identified by clinic notes and correspondence as being potential recruits, and were sent the patient information sheets. Of those screened, 284 were deemed eligible, giving a ‘screen positive’ rate of 37%. The numbers screened at individual centres varied between 14 and 399; the percentage of eligible women recruited varied between 55% and 100%.

To explore why ‘screen positive rates’ may have varied across sites, we asked all 11 screeners from the seven sites to respond to a series of 20 identical vignettes. These were mainly based on actual GP referral letters. Sixteen vignettes mentioned one or more definite inclusion criteria; the remainder had possible inclusions. Four had definite exclusions; 15 had possible exclusions. Free text comments were sought to clarify the screeners' decisions.

For six vignettes everyone agreed that the patient was eligible; for one all agreed she was not eligible; the breakdown for the remainder was mixed. Comments amplified decisions. The findings indicated that inclusion and exclusion criteria should be more explicit and that where information is missing from referral letters, the default action should be to invite the woman to attend for detailed screening. Vignettes could usefully be used as training tools for those screening and consenting patients.

